# Commitment of Autologous Human Multipotent Stem Cells on Biomimetic Poly-L-Lactic Acid-Based Scaffolds Is Strongly Influenced by Structure and Concentration of Carbon Nanomaterial

**DOI:** 10.3390/nano10030415

**Published:** 2020-02-27

**Authors:** Marika Tonellato, Monica Piccione, Matteo Gasparotto, Pietro Bellet, Lucia Tibaudo, Nicola Vicentini, Elisabetta Bergantino, Enzo Menna, Libero Vitiello, Rosa Di Liddo, Francesco Filippini

**Affiliations:** 1 Department of Biology, University of Padua, 35131 Padua, Italy; marika.tonellato@gmail.com (M.T.); pietro.bellet@studenti.unipd.it (P.B.); lucia.tibaudo@unipd.it (L.T.); elisabetta.bergantino@unipd.it (E.B.); libero.vitiello@unipd.it (L.V.); 2 Department of Pharmaceutical and Pharmacological Sciences, University of Padua, 35131 Padua, Italy; monica.piccione@studenti.unipd.it; 3 Department of Biomedical Sciences, University of Padua, 35131 Padua, Italy; 4 Department of Chemical Sciences, University of Padua, 35131 Padua, Italy; nicola.vicentini@unipd.it (N.V.); enzo.menna@unipd.it (E.M.); 5 Interuniversity Institute of Myology (IIM), Italy; 6 Inter-departmental Research Center for Myology (CIR-Myo), University of Padua, 35131 Padua, Italy

**Keywords:** biomimetic nanomaterials, human circulating multipotent cells, PLLA-based scaffolds, carbon nanostructures, carbon nanotubes, carbon nanohorns, graphene, MYOD1, myogenic commitment

## Abstract

Nanocomposite scaffolds combining carbon nanomaterials (CNMs) with a biocompatible matrix are able to favor the neuronal differentiation and growth of a number of cell types, because they mimic neural-tissue nanotopography and/or conductivity. We performed comparative analysis of biomimetic scaffolds with poly-L-lactic acid (PLLA) matrix and three different *p*-methoxyphenyl functionalized carbon nanofillers, namely, carbon nanotubes (CNTs), carbon nanohorns (CNHs), and reduced graphene oxide (RGO), dispersed at varying concentrations. qRT-PCR analysis of the modulation of neuronal markers in human circulating multipotent cells cultured on nanocomposite scaffolds showed high variability in their expression patterns depending on the scaffolds’ inhomogeneities. Local stimuli variation could result in a multi- to oligopotency shift and commitment towards multiple cell lineages, which was assessed by the qRT-PCR profiling of markers for neural, adipogenic, and myogenic cell lineages. Less conductive scaffolds, i.e., bare poly-L-lactic acid (PLLA)-, CNH-, and RGO-based nanocomposites, appeared to boost the expression of myogenic-lineage marker genes. Moreover, scaffolds are much more effective on early commitment than in subsequent differentiation. This work suggests that biomimetic PLLA carbon-nanomaterial (PLLA-CNM) scaffolds combined with multipotent autologous cells can represent a powerful tool in the regenerative medicine of multiple tissue types, opening the route to next analyses with specific and standardized scaffold features.

## 1. Introduction

The challenges that regenerative medicine faces depend on the complexity of the tissue and organs to be repaired. In neural regenerative medicine, repair strategies must consider whether the damage has impaired either peripheral nerves or the central nervous system (CNS), which consists of the brain, cerebellum, and spinal cord. While peripheral neurons are endowed with remarkable regeneration capacity that allows functional recovery [1], CNS injury is more severe because central neurons are unable to restore correct axonal and dendritic connections. The deep negative effects on quality of life, the huge number of affected people, and the financial burden caused by spinal-cord and traumatic brain injuries are attracting much attention from society and result in significant efforts from the scientific community [2,3] to address this issue.

Besides nerve-tissue impairment, another cause of lifelong functional deficit is volumetric muscle loss, which happens whenever the extent of tissue damage and/or removal overwhelms the skeletal muscle’s regenerative capabilities. Neither transplant nor other traditional medicine approaches can solve this problem, at least so far, thus prompting muscle-tissue engineering research as a promising solution for these issues. Moreover, the coordinated regeneration of muscle and nerves is needed because of their complex interconnection, mediating motor co-ordination [4]. 

Current therapeutic strategies focus on the culture and differentiation of stem cells to repopulate an injury site. Given their availability, potency, possibility of autologous implant, and relative lack of ethical concerns, adult stem cells are of special interest to regenerative medicine. However, fast and reliable differentiation protocols are needed to make their application more accessible. With respect to embryonic and induced pluripotent stem cells showing complete tissue-regeneration capacity, adult stem cells are multipotent, i.e., able to differentiate towards a limited number of lineages. Mesenchymal stem cells (MSCs) and fat-derived stem cells are widely used for regenerative-medicine purposes [5,6,7]. Among blood-derived stem cells, human circulating multipotent stem cells (hCMCs) [8] represent an interesting model as they can differentiate into progenitors for several mesenchymal tissue types, such as adipocytes, osteoblasts, chondroblasts, or muscle cells in response to different culture conditions. These cells are extremely sensitive to changes in the microenvironment, and respond to biomimetic stimuli by sudden variations in gene expression; importantly, when specific growth factors are added, they can also commit to neuronal differentiation [9]. 

When cells are seeded onto scaffolds with a given set of microenvironmental properties, they integrate physical and chemical inputs to provide a coherent and coordinated response. Changes in substrate stiffness can induce the osteogenic differentiation of MSCs seeded onto polyacrylamide gels [10], and balance the ratio between astrocytic and neuronal lineages in neuronal-stem-cell differentiation [11,12]. Similarly, conductive reduced graphene oxide (RGO) hydrogels can enhance the myogenic-gene expression of myoblasts, boosting their differentiation [13], while multiwalled carbon-nanotube (CNT) nanocomposites were found to enhance the neuronal differentiation of neural stem cells [14].

In most cases, the biomimetic potential of scaffolds resides in their capacity to recapitulate physiological mechanotransduction signals by mimicking the stiffness and roughness of the original tissue, thus properly supporting cell growth, or to mediate differentiation. In neural regenerative medicine, however, scaffold conductivity is also important because of the special nature of the electric signaling that characterizes neurons. Conductive CNTs represent an established nanofiller for boosting neuronal commitment and differentiation [15,16,17,18,19,20]. FDA-approved poly(L-lactic) acid (PLLA) is widely used in regenerative medicine, and scaffolds consisting of CNTs dispersed in a PLLA matrix (CNT@PLLA) were able to commit hCMCs toward a neuronal lineage even in the absence of exogenous neurotrophins, highlighting their biomimetic potential [8].

Even though CNTs are most commonly used, emerging evidence suggests that other carbon nanomaterials (CNMs) can be used in neuronal regenerative medicine. In particular, graphene-based materials are receiving ever-growing attention because of their lower cost and increased biocompatibility [21,22,23,24,25,26,27,28]. For regenerative-medicine purposes, the cytotoxicity of different CNMs [29,30,31,32,33,34,35] is the first point to be addressed. Common strategies to achieve this goal include: (i) improved purification protocols for limiting the presence of toxic residuals such as heavy metals [35]; (ii) CNM functionalization [35,36,37,38] to improve their solubility in the matrix and, once released by progressive matrix biodegradation, to avoid cell death caused by CNM uptake and intracellular aggregation [39,40]; (iii) use of CNMs as nanofillers at low concentration, dispersed into various biocompatible polymer matrices [41,42,43,44,45]. This latter strategy brought the development of a number of different nanocomposite scaffolds in which the polymer itself acts as the scaffold–cell interface and may provide further stimuli [46,47,48,49]. 

We recently used PLLA composite scaffolds based on *p*-methoxyphenyl functionalized CNTs (CNT@PLLA), either flat or electrospun, to promote neuronal growth and differentiation starting from either SH-SY5Y cells or hCMCs [8,50,51]. Given that different CNMs may vary in the individual weight of each cue they can provide (electrical, chemical, mechanical, and topological), we developed novel PLLA composite scaffolds with CNM fillers showing different nanotopography and conductivity. Specifically, we used CNTs, RGO, and carbon nanohorns (CNHs), all of which underwent a detailed physicochemical characterization and proved to be fully compatible with neuronal-cell growth at concentrations of up to 5% [52]. Mechanical properties were found to be highly influenced by fillers, as ductility was improved with respect to bare PLLA for nanofiller concentrations in the range of 0.25–1 wt% [52]. Electrical percolation was not found to take place in CNH composites, with CNT@PLLA showing a sharp increase in conductivity at a lower CNM concentration with respect to RGO@PLLA. The extent of conductivity after the percolation threshold was also significantly higher for the former as compared to for the latter [52]. 

In order to shed more light on the individual contribution of nanotopography and conductivity stimuli to cell fate commitment, we performed comparative analysis of these newly developed scaffolds, aimed at comparing well-established CNT-based scaffolds to the emerging graphene nanofiller and to CNHs, which so far have not received significant attention in regenerative medicine. Moreover, we aimed at investigating how the variation of multiple topological stimuli onto hCMCs could eventually result in their commitment towards cell lineages other than neuronal.

In these experiments, we found that less conductive scaffolds, i.e., bare PLLA, CNH- and RGO-based nanocomposites enhance the expression of myogenic marker genes in hCMCs, while they are not effective on myoblast-to-myotube differentiation.

## 2. Materials and Methods 

### 2.1. Nanofiller Feature, and Scaffold Preparation and Sterilization

Scaffolds were synthesized as previously described [50,51]. Multiwalled CNTs (from SouthWest NanoTechnologies Inc., Norman, OK, USA) had the following dimensions: outer diameter 10 ± 1 nm, internal diameter 4.5 ± 0.5 nm, lengths 3–6 µm. CNHs (from Carbonium s.r.l., Padova, Italy) presented a dahlia-type shape with a diameter of 60–120 nm. RGO powder (from ACS Material, LLC, Pasadena, CA, USA) had lateral dimensions between 1 and 2 µm, and flakes consisted of few irregularly overlapping layers with many corrugations. Chemical modification and purification of the CNMs was performed through diazonium-based reactions, as previously described [52], yielding *p*-methoxyphenyl-functionalized derivatives (CNM-PhOMe) with improved dispersibility. CNM@PLLA blend solutions in CHCl_3_ were prepared by adding a dispersion of CNM-PhOMe in chloroform obtained via sonication to a chloroform solution of PLLA (6 wt%) under continuous stirring. Each CNM-PhOMe was dispersed within the various blends at 0.25 and 1 wt% (with respect to polymer content). Scaffold films were obtained through solvent evaporation at 50 °C. Functionalized CNM@PLLA films were characterized as reported [52].

In experiments with cells, scaffolds were cleaned with 70% ethanol, sterilized under UV radiation for 90 minutes, and incubated for 3 h with Dulbecco’s Modified Eagle Medium/Nutrient Mixture F-12 (DMEM/F-12) GlutaMAX™ supplement (Invitrogen, Milan, Italy) supplemented with 10% heat-inactivated fetal bovine serum (FBS, Euroclone, Milan, Italy). In the control samples, culture wells were coated with 0.005% gelatine (porcine skin, Sigma-Aldrich, Milan, Italy)/1µg/ml poly-L-lysine (Invitrogen).

### 2.2. SH-SY5Y, Primary Myoblast, and Adipocyte Culture 

Exponentially growing human neuroblastoma-derived SH-SY5Y cells were cultured with DMEM/F-12 GlutaMAX™ supplement (Invitrogen), 10% FBS (Euroclone), and 25 μg/ml of gentamicin (Sigma-Aldrich) (SH-SY5Y growth medium), in a humidified atmosphere of 5% CO_2_ in air at 37 °C. Cells were maintained by sub-culturing 1·10^6^ cells into 25 cm² flasks (Sarstedt) every 2 days (once 80% confluence was reached). 

Immortalized human myoblasts, obtained by double transduction with hTERT and cdk4, were kindly supplied by the Institut de Myologie (Pitié-Salpétrière Hospital, Paris, France). These myoblasts (*wt AB1190*) were cultured with a growth medium containing F12 supplemented with 20% FBS (Invitrogen), 25 µg/ml fetuin (Invitrogen), 5 ng/ml hEGF (ImmunoTools GmbH, Friesoylthe, Germany), 0.5 ng/ml bFGF (ImmunoTools), 5 µg/ml insulin (Sigma-Aldrich), and 0.2 µg/ml dexamethasone (Sigma-Aldrich). In the scaffold experiments, cells were seeded onto CNM@PLLA scaffolds and gelatin-coated control wells at 25k cells/well density. On the sixth day of culturing, medium was replaced with the differentiation medium (DMEM and 10 µg/ml insulin) in the absence of FBS. 

Human adipose tissue from abdominal dermolipectomy was used to obtain primary adipocyte cultures. Briefly, samples were digested with 2 mg/mL collagenase A (Roche Diagnostics, Mannheim, Germany) in Dulbecco’s modified essential medium F12 (DMEM-F12; Sigma-Aldrich) for 45 min at 37 °C. Digested tissue was filtered through a 100 µm nylon membrane to eliminate undigested fragments. After centrifugation (1200 rpm for 10 min), cells from the stromal vascular fraction of the adipose tissue were collected and kept at –20 °C until use.

### 2.3. hCMC Isolation, Culture, and Differentiation

hCMCs were isolated from human peripheral blood under Italian Ethics Committee authorization and informed consent as previously described [8]. Cells were seeded in a density of 10^4^ cells/cm^2^, with Minimum Essential Medium Alpha (αMEM) supplemented with 16.5% heat-inactivated fetal bovine serum (FBS; Invitrogen), 50 U/ml penicillin (Invitrogen Life Technologies), 50 μg/ml streptomycin (Invitrogen Life Technologies), and 1% L-glutamine (Sigma-Aldrich) (hCMC growth medium). For the scaffold experiments, cells were seeded onto the CNM@PLLA scaffolds or coated wells at 2 × 10^4^ cells/cm^2^ density in DMEM/F-12 (Invitrogen) supplemented with 16.5% FBS, 50 U/ml penicillin, 50 μg/ml streptomycin (day –1). Induction started after 24 h (day 0), when the medium was replaced by DMEM/F-12 supplemented with 2% FBS (low FBS medium). In parallel, cells under proliferative conditions (i.e., in the growth medium) were used as controls.

### 2.4. Morphological Analysis

Myoblasts were stained with 2 µM Calcein-AM (Biotium Inc., Fremont, CA, USA) in Hank’s Balanced Salt Solution (HBSS, Invitrogen) and 10µg/ml Hoechst 33258 (Invitrogen) for 45 min in the dark at 37 °C and 5% CO_2_. Cells were visualized under a Leica DM4000B fluorescent microscope using a GFP and DAPI filter. Fusion index, which describes the number of nuclei inside myotubes as a percentage of the total number of nuclei, was evaluated with Fiji [53].

### 2.5. RNA Extraction and qPCR

RNA from each sample was obtained after cell homogenization with TRIzol® reagent (Invitrogen) according to the manufacturer’s protocol. In order to reduce noise from cells growing at the bottom of the well, scaffolds were moved to a new 24 well plate prior to adding TRIzol®. RNA was dried and dissolved in RNase-free water. RNA was quantified by measuring absorbance at 260 nm with NanoDrop2000 (ThermoFisher Scientific, Milan, Italy).

Reverse transcription was performed with GoScript™ Reverse Transcription System (Promega Italia S.r.l., Milan, Italy) using only oligo(dT)_15_ primers, while the amplification reaction was carried out using SensiFAST SYBR No-ROX Kit (Bioline, Aurogene S.r.l., Rome, Italy) and a Rotor-Gene 3000 thermal cycler (Corbett Research-Qiagen Italy S.r.l., Milan, Italy). At least three independent experiments were performed in duplicate. The housekeeping control for normalization was ribosomal protein S13 [50,54]. The comparative CT method (2^-ΔCt^) was used to quantify gene-expression levels. Primer pairs (Invitrogen and Sigma-Aldrich) were the following (A, amplicon; F, forward; R, reverse):

- Leptin, NM_000230.2, A: 259bp,
  F: CCATAACAGCCAACAGGTG, R: CCTCTCGCTGTAACTCACTGC;
- MAP2, NM_002374.3, A: 253bp,
  F: ATAGACCTAAGCCATGTG, R: GGGACTGTGTAATGATCTC;
- MYOD1, NM_000230.2, A: 269bp,
  F: GAGGCGGGAGAACTGAAG, R: CTGCTACATTTGGGACCG;
- MYOG, NM_002479.6, A: 259bp,
  F: GGACAGCATCACAGTGGAAG, R: GAATGAGGGCGTCCAGTC;
- Nestin, NM_006617.1, A: 257bp,
  F: CAGGGGAGGACTAGGAAAAGA, R: GAGATGGAGCAGGCAAGAG;
- Pax7, NM_001135254.2, A: 259bp,
  F: CTTGAGAACAGGACGGGTC, R: GTCTTGGTTTTGGTGCCTC;
- Plin1, NM_002666.5, A: 242bp,
  F: CACAGCCACATTTCCATTTG, R: CAATGAAGGGGAACAGGG;
- Ribosomal protein S13 (S13), NM_001017.2, A: 259bp,
  F: TACAAACTGGCCAAGAAGGG, R: GGTGAATCCGGCTCTCTATTAG;
- TUBß3, NM_001197181, A: 259bp,
  F: AGGAAGAGGGCGAGATGTA, R: CAATAAGACAGAGACAGGAGCAG.

### 2.6. Immunofluorescence

Immortalized human myoblasts were fixed in 2% paraformaldehyde (PFA) for 15 minutes and permeabilized in 0.5% Triton X-100 (Sigma-Aldrich) in phosphate buffer solution (PBS). After that, samples were blocked in 10% goat serum (Sigma-Aldrich) in PBS for 45 minutes at 20 °C (room temperature). Cells were stained overnight at 4 °C using MyoD primary antibody (554130, BD Biosciences, Milan, Italy) diluted in 3% bovine serum albumin (BSA) (Sigma-Aldrich). The following day, the secondary antibody used for detection was Alexa Fluor 488 (Molecolar Probes-ThermoFisher) diluted in 3% BSA for 1 h at 20 °C (room temperature). A Leica DMR5000 microscope was used for image acquisition.

### 2.7. Statistical Analysis

Statistical analysis was performed using two-way ANOVA, and results were considered significant when *p* < 0.05.

## 3. Results and Discussion

### 3.1. Scaffold Effect on Neuronal Differentiation

**Figure 1:**
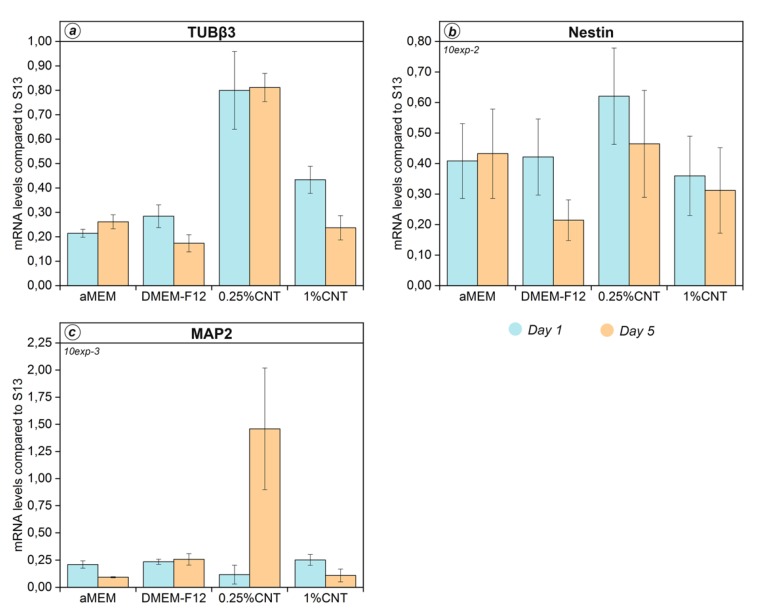
Modulation of (**a**) Tubß3, (**b**) nestin, and (**c**) MAP2 during first five days after differentiation. αMEM: proliferative medium w/o scaffold; DMEM-F12: differentiative medium w/o scaffold; 0.25%CNT: 0.25 wt% *p*-methoxyphenyl functionalized carbon nanotubes (CNT@PLLA); 1%CNT: 1 wt% CNT@PLLA. Data represent mean ± SEM of three independent experiments performed in duplicate.

In a preliminary set of experiments, we followed our previous protocols [8] to check how variation in nanofiller type and concentration influences gene expression in hCMCs. Cells were grown in differentiative medium and seeded onto CNT@PLLA, RGO@PLLA, and CNH@PLLA at either 0.25 or 1 wt%, on bare PLLA, and, as control, in wells without scaffolds. As an additional control without scaffold, cells were grown into a proliferative medium. Early expression of neuronal marker genes Nestin, Tubß3, and MAP2 was analyzed (days 1 and 5 from induction, see Materials and Methods). When expression profiles were compared to previous evidence [8], only data from control samples were found to be congruent. In particular, observed changes in the expression when shifting from proliferative to differentiative medium were confirmed with all three marker genes. The fact that expression profiles from CNT@PLLA-treated samples were not confirmed (see Figure 1) pointed at scaffolds as the likely cause of this variability. In order to clarify this issue, we analyzed individual replicates, and random variation was observed only in the expression profiles of cells seeded onto scaffolds. Such variability also concerned cells cultured onto RGO@PLLA and CNH@PLLA scaffolds (Figure S1).

**Figure 2:**
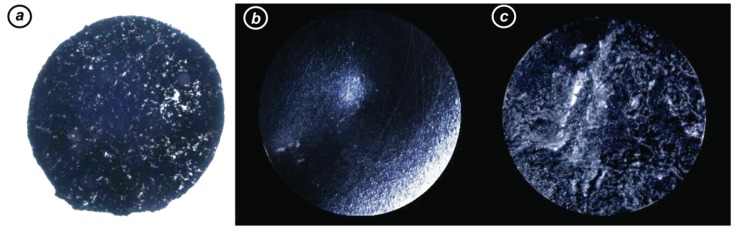
Examples of batch-to-batch variability of scaffold produced manually as described: (**a**) 0.25 wt% CNT@PLLA with nonuniform dispersion as seen with transmission microscopy. White spots were caused by light passing through scaffold because of very low nanofiller concentration; 1 wt% CNT@PLLA with (**b**) valley and hill morphology or (**c**) variable roughness as seen by stereomicroscopy. Here, white spots are reflexes highlighting surface imperfections.

Indeed, hCMCs and other multipotent cells are highly responsive to even minor changes in local stimuli; thus, we wondered if inhomogeneity of nanotopographic and conductive features of scaffolds might have accounted for the observed results. Scaffold inspection by both transmission and stereomicroscopy (visible light) unveiled higher local variation (both in terms of dispersion and surface homogeneity) than the previously characterized ones [8,50,51,52]. Figure 2 shows examples of highly variable scaffolds.

In particular, we observed subregions (Figure 2a) in which nanofiller concentration was clearly different, lower in the translucent parts and increased in the darker ones. Considering that minor fluctuations in CNM concentration in the 0.25–1 wt% range corresponded to steep variation (up to 10^6^-fold) in resistivity, cells seeded on scaffold parts with such different conductivities would receive many different stimuli. Variation in Young’s modulus is also likely to occur. Moreover, surface irregularities were observed (Figure 2b,c). Concave and convex areas have an effect on seeding: cell density was increased in valleys and decreased in hills. In turn, this could result in providing hCMCs with uncontrolled and unpredictable commitment signals. 

### 3.2. Selection of Non-Neuronal Markers

Neural commitment is often inferred through the combined modulation profile of nestin, Tubß3, and MAP2. However, recent evidence showed that these genes are also expressed in cell types other than neurons [55,56], and this may account for altered profiles when multiple differentiation pathways occur in the same sample. In this context, we considered further and more specific qRT-PCR markers of which the expression could demonstrate the eventual differentiation of hCMCs towards non-neuronal tissue. Among the possible lineages to which hCMCs have the ability to commit, we considered myogenic and adipogenic ones. The following marker genes were identified: (i) PAX7 (NM_001135254.2), which is expressed during the early stages of myogenic commitment up to early myoblast differentiation; (ii) MYOD1 (NM_002478.5), of which the expression, although progressively dropping, remains high until late myoblast differentiation; (iii) MYOG (NM_002479.6), which is expressed at high levels during late myogenesis; (iv) leptin (NM_000230.3), which acts in transcriptional regulation in early white adipocyte differentiation; and (v) PLIN1 (NM_001145311.2), expressed at high levels in mature adipocytes. Primers reported in the Section 2.5 were designed, and their specificity was tested using adipocytes, myoblasts, and myotubes as positive controls (not shown). 

### 3.3. Scaffold Effect on Marker-Gene Modulation

**Figure 3:**
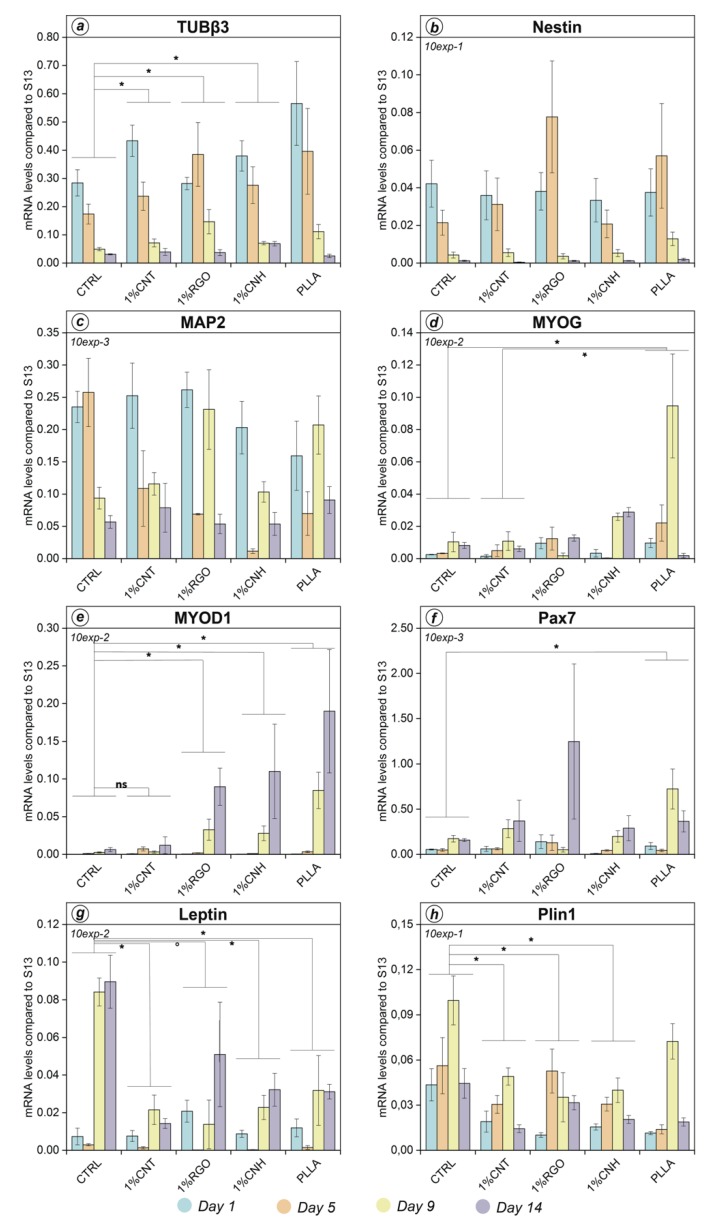
Marker-gene expression during 14 day treatment. Profiled genes in panels a to h are indicated in the upper horizontal bar of each panel. CTRL: differentiative medium w/o scaffold; 1%CNT: 1 wt% CNT@PLLA; 1%RGO: 1 wt% RGO@PLLA; 1%CNH: 1 wt% CNH@PLLA; PLLA: bare PLLA. Control samples were grown in same medium but without scaffolds. Data represent mean ± SEM of three independent experiments performed in duplicate. ° Significance at *p* < 0.1 among indicated samples; * significance at *p* < 0.05 among indicated samples.

In order to minimize local scaffold variability, only 1 wt% CNM@PLLA scaffolds were used in further qRT-PCR experiments. Expression was profiled at days 1, 5, 9, and 14 from differentiation (see Materials and Methods), so that we could verify the presence of any modulation of the neuronal markers, even over the first five days, and possibly capture any modulation of the new ones. Figure 3 shows that, on day 1, nestin expression was comparable with the control in every tested condition, as confirmed by statistical analysis (ANOVA). Interestingly, nestin expression seemed to be higher during the early time points. This might depend on a boost in neural differentiation, but induction towards other lineages might contribute as well: as reported in the Human Protein Atlas (HPA), nestin expression is even higher in muscle tissue than in the brain (Figure S2). Instead, TUBβ3 expression showed a progressive decrease, compatible with commitment towards multiple lineages (Figure 3a). MAP2 modulation appeared unclear, as statistical analysis did not show meaningful difference between samples or time points (Figure 3c). This likely depended on the magnified effects of scaffold inhomogeneity on the overall very low expression of MAP2. Myogenic marker MYOD1 was barely expressed in the control and in the 1 wt% CNT@PLLA scaffolds, whereas other conditions showed strong, albeit variable, expression at days 9 and 14 (Figure 3e). Except for CNT@PLLA, during the late time points from induction, MYOD1 mRNA levels showed roughly a one-order-of-magnitude increase in parallel with electrical resistivity. MYOD1 is quite specific for myogenic lineage, as shown by HPA data (Figure S2). MYOG also showed the highest expression in cells grown onto the less conductive scaffold (bare PLLA). Independent of the CNM nanofiller, scaffolds did not seem to favor adipogenic commitment, as PLIN1 and leptin expression in the scaffold samples was indeed similar to or even lower than in the control. 

A certain percentage of myogenic commitment in hCMC population might also explain peculiar expression profiles for nestin, whose high levels during the early time points could anticipate later modulation in MYOD1 and PAX7. Great variability between the biological replicates more likely reflected scaffold imperfections and hCMC multipotency, while in previous experiments, some uncertain values in 0.25 wt% samples could have been caused by the presence of small areas almost devoid of nanofiller (nearly bare PLLA). Finally, in addition to already committed cells, multipotent-to-oligopotent intermediates might be present as well.

### 3.4. Production and Subcellular Localization of MyoD1 Protein in hCMCs and Control Myoblasts

**Figure 4:**
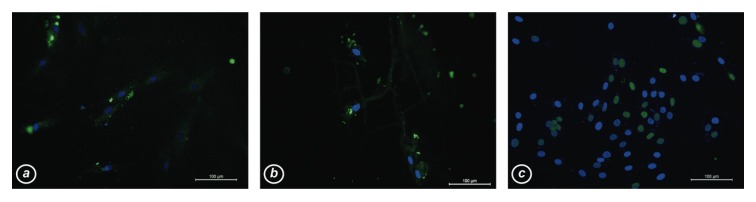
MyoD1 subcellular localization. (**a**) Human circulating multipotent stem cells (hCMCs) grown onto 1 wt% CNH@PLLA for 9 days; (**b**) hCMC grown onto 1 wt% CNH@PLLA for 14 days; (**c**) myoblasts seeded on well.

In order to assess whether increased MYOD1 mRNA expression could actually result in the production of the corresponding protein, and thus the activation of downstream myogenic genes, we performed immunofluorescence analysis. In hCMCs grown onto 1 wt% CNH@PLLA scaffolds for 9 or 14 days (Figure 4a,b), faint or punctuated MyoD1 signal (green) could be observed at subcellular localization other than in the nuclei (blue). Instead, in the positive control (myoblasts grown onto the well bottom, Figure 4c) MyoD1 colocalized at the nuclei. Since phosphorylation of MyoD1 at its C-terminal domain is required for translocation into the nucleus [57], one could not rule out that MyoD1 was actually produced in the analyzed hCMCs, but was unable to translocate to the nucleus as regulating factors (e.g., kinases) were not yet expressed in these culture conditions. Further studies will be needed to verify this hypothesis.

### 3.5. Effect of CNM@PLLA Scaffolds on Myoblast Differentiation

**Figure 5:**
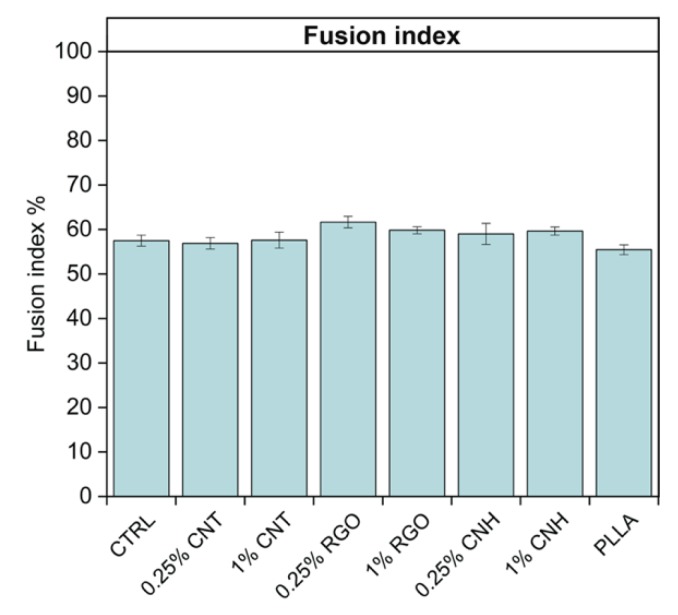
Effect of the different scaffolds on myoblasts fusion during differentiation towards myotubes. CTRL: differentiative medium w/o scaffold; 0.25–1%CNT: 0.25–1% wt CNT@PLLA; 0.25–1%RGO: 0.25–1% wt RGO@PLLA; 0.25–1%CNH: 0.25–1% wt CNH@PLLA; PLLA: bare PLLA. Data are shown as mean ± SEM.

In previous works with CNT-based scaffolds and biomimetic peptides [8,50,51], it emerged that scaffolds are effective in early cell fate commitment and poor in modulating subsequent differentiation steps, while biomimetic peptides reproducing guidance cues do the opposite. Since this was reported with neuronal commitment, we investigated whether scaffolds that can commit towards myogenic lineage were similarly limited or could eventually boost further myoblast differentiation. Therefore, we tested the scaffold effects on myoblast-to-myotube transition using the fusion index as an indicator. Figure 5 shows that, under the culture conditions for neuronal differentiation, scaffolds were not active in boosting myoblast differentiation (no statistically meaningful difference among samples when using ANOVA).

## 4. Conclusions

Previous evidence suggested that highly conductive CNTs boost neuronal differentiation [8], while this work showed that less conductive CNH@, RGO@PLLA, and PLLA scaffolds enhance the expression of myogenic markers. In addition, we found that local variations in nanofiller dispersion and surface homogeneity, and thus in conductivity and nanotopography, are likely to mediate contemporary commitment towards multiple lineages and potency stages. When considering regenerative medicine perspectives, in particular with regard to implants, local variation in scaffold composition is an issue to be solved by alternatively following up lab-scale production by postproduction quality assessment, or by shifting to industrial standards.

Evidence that scaffolds are good at mediating commitment to the myogenic lineage and poor at further modulating myoblast differentiation towards myotubes agrees with previous results with CNT-based scaffolds. Indeed, scaffold nanotopography and conductivity provide tissue identity, biomimetic nanomaterial signaling to multipotent cells in order to provide them with the right commitment, while guidance cues are expected to be effective once their targets are exposed at the differentiated cell surface.

## Supplementary Materials

The following files are available online at https://www.mdpi.com/2079-4991/10/3/415/s1. Figure S1: Modulation of neuronal markers; Figure S2: Modulation of non-neuronal markers according to the Human Protein Atlas.
