# Supplementary material for: Commitment of Autologous Human Multipotent Stem Cells on Biomimetic Poly-L-Lactic Acid-Based Scaffolds Is Strongly Influenced by Structure and Concentration of Carbon Nanomaterial
Source: Nanomaterials (Basel). 2020 Feb 27;10(3):415. doi: 10.3390/nano10030415 (PMC7152835; doi:10.3390/nano10030415)
Supplement: Supplementary file 1 [file nanomaterials-10-00415-s001.pdf]

Supplementary information to manuscript:

Commitment of Autologous Human Multipotent Stem Cells on Biomimetic Poly-L-lactic Acid-Based Scaffolds Is Strongly Influenced by Structure and Concentration of Carbon Nanomaterial

Marika Tonellato [1#], Monica Piccione [2#], Matteo Gasparotto[1*], Pietro Bellet[1], Lucia Tibaudo[1,3], Nicola Vicentini[4], Elisabetta Bergantino[1], Enzo Menna[4], Libero Vitiello[1,5,6], Rosa Di Liddo[2*] and Francesco Filippini[1*]

1 Department of Biology, University of Padua, 35131 Padua, Italy; marika.tonellato@gmail.com (M.T.); pietro.bellet@studenti.unipd.it (P.B.); lucia.tibaudo@unipd.it (L.T.); elisabetta.bergantino@unipd.it (E.B.); libero.vitiello@unipd.it (L.V.)

2 Department of Pharmaceutical and Pharmacological Sciences, University of Padua, 35131 Padua, Italy; monica.piccione@studenti.unipd.it

3 Department of Biomedical Sciences, University of Padua, 35131 Padua, Italy.

4 Department of Chemical Sciences, University of Padua, 35131 Padua, Italy; nicola.vicentini@unipd.it (N.V.); enzo.menna@unipd.it (E.M.)

5 Interuniversity Institute of Myology (IIM), Italy

6 Inter-departmental Research Center for Myology (CIR-Myo), University of Padua, 35131 Padua, Italy

# These authors contributed equally to this work.

* Correspondence: matteo1.gasparotto@gmail.com (M.G.); rosa.diliddo@unipd.it (R.D.L.); francesco.filippini@unipd.it (F.F.)

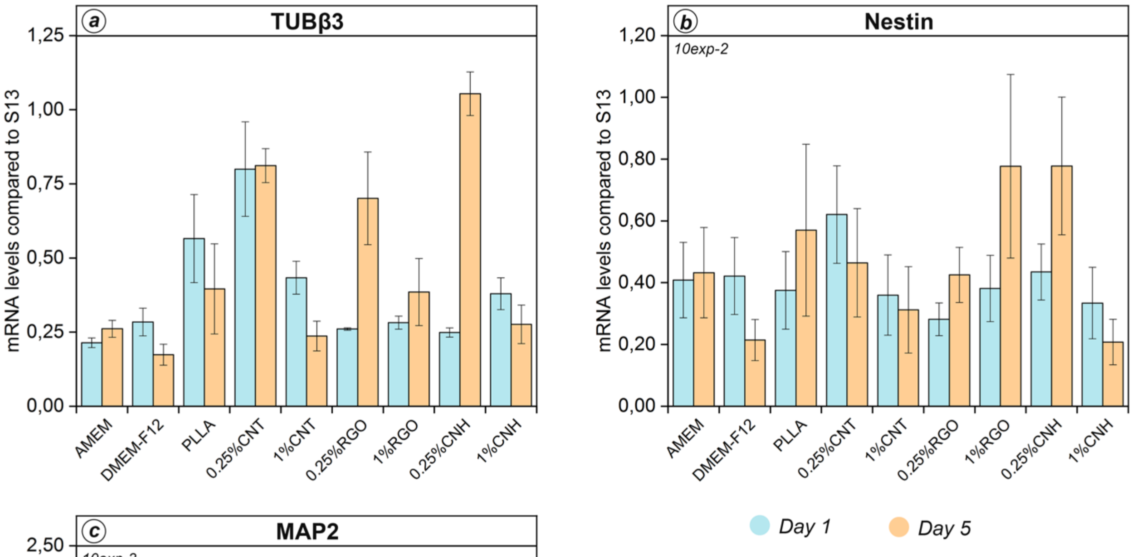

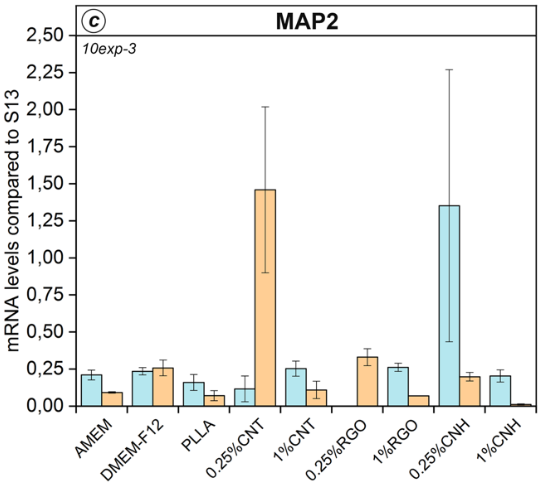

Figure S1. Modulation of (**a**) Tubß3, (**b**) Nestin and (**c**) MAP2 during the first five days after differentiation. AMEM: proliferative medium w/o scaffold; DMEM-F12: differentiative medium w/o scaffold; PLLA: bare PLLA; 0.25% and 1%CNT: 0.25% and 1%w/w CNT@PLLA respectively; 0.25% and 1%RGO: 0.25% and 1%w/w RGO@PLLA respectively; 0.25% 1%CNH: 0.25% and 1%w/w CNH@PLLA. Data represent the mean ± SEM of three independent experiments performed in duplicate

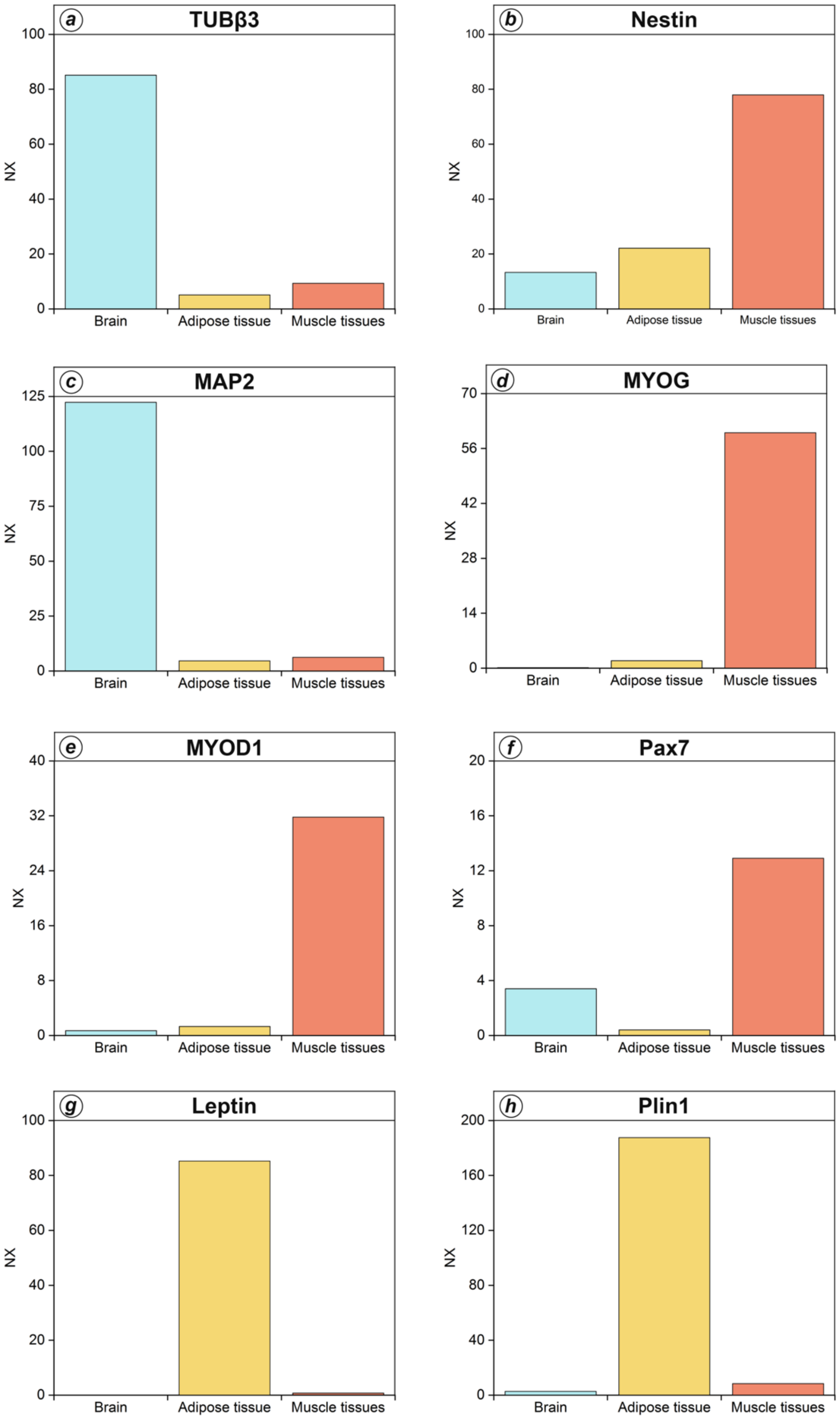

Figure S2. Expression levels of marker genes used in qRT-PCR analysis as reported in the Human Protein Atlas.
